# Aflatoxin exposure during the first 36 months of life was not associated with impaired growth in Nepalese children: An extension of the MAL-ED study

**DOI:** 10.1371/journal.pone.0172124

**Published:** 2017-02-17

**Authors:** Nicole J. Mitchell, Hui-Husan Hsu, Ram Krishna Chandyo, Binob Shrestha, Ladaporn Bodhidatta, Yu-Kang Tu, Yun-Yun Gong, Patricia A. Egner, Manjeswori Ulak, John D. Groopman, Felicia Wu

**Affiliations:** 1 Department of Food Science and Human Nutrition, Michigan State University, East Lansing, Michigan, United States of America; 2 Institute of Epidemiology & Preventive Medicine, College of Public Health, National Taiwan University, Taipei, Taiwan; 3 Centre for Intervention Science in Maternal and Child Health, Centre for International Health, University of Bergen, Bergen, Norway; 4 Department of Child Health, Institute of Medicine, Tribhuvan University, Kathmandu, Nepal; 5 Walter Reed/Armed Forces Research Institute of Medical Sciences, Research Unit, Kathmandu, Nepal; 6 Department of Enteric Diseases, Armed Forces Research Institute of Medical Sciences, Bangkok, Thailand; 7 Institute for Global Food Security, School of Biological Sciences, Queen’s University Belfast, Belfast, United Kingdom; 8 Department of Environmental Health Sciences, Johns Hopkins University, Bloomberg School of Public Health, Baltimore, Maryland, United States of America; University of Liverpool, UNITED KINGDOM

## Abstract

Exposure to aflatoxin, a mycotoxin common in many foods, has been associated with child growth impairment in sub-Saharan Africa. To improve our understanding of growth impairment in relation to aflatoxin and other risk factors, we assessed biospecimens collected in Nepalese children at 15, 24, and 36 months of age for aflatoxin exposure. Children (N = 85) enrolled in the Bhaktapur, Nepal MAL-ED study encompassed the cohort analysed in this study. Exposure was assessed through a plasma biomarker of aflatoxin exposure: the AFB_1_-lysine adduct. The aflatoxin exposures in the study participants were compared to anthropometrics at each time period (length-for-age [LAZ], weight-for-age [WAZ], and weight-for-length [WLZ] z-scores), growth trajectories over time, age, and breastfeeding status. Results demonstrated chronic aflatoxin exposure in this cohort of children, with a geometric mean of 3.62 pg AFB_1_-lysine/mg albumin. However, the chronic aflatoxin exposure in this cohort was not significantly associated with anthropometric z-scores, growth trajectories, age, or feeding status, based on the available time points to assess aflatoxin exposure. Low mean levels of aflatoxin exposure and infrequent occurrence of stunting, wasting, or underweight z-score values in this cohort are possible contributing factors to a lack of evidence for an association. Further research is needed to examine whether a threshold dose of aflatoxin exists that could induce child growth impairment.

## Introduction

For over 50 years, the dietary mycotoxin (fungal toxin) *aflatoxin* has been known to cause liver cancer in experimental models and in people; but only more recently has interest grown in whether aflatoxin also impairs the growth of children. The most potent naturally occurring chemical liver carcinogen, aflatoxin is produced primarily by the fungi *Aspergillus flavus* and *A*. *parasiticus*, which frequently affect crops in warm climates. Aflatoxin contaminates staple foods such as maize and peanuts, resulting in chronic, substantial exposure in populations with uniform diets focused primarily around maize- and peanut-based foods. In addition to its carcinogenic effects, aflatoxin exposure has been associated with immune dysfunction, growth faltering, and–at high doses–death from acute liver failure [1–3]. Dietary aflatoxin exposure is well documented in tropical climate zones such as sub-Saharan Africa and Southeast Asia. However, the distribution of aflatoxin-contaminated foods may extend to sub-tropical and even temperate climate zones, due to international food trade and climate change [4,5].

In recent years, epidemiological studies have examined the effects of aflatoxin exposure on early child development. It is well known and documented that aflatoxin exposure results in growth impairment in several animal species. Clinical and pathological signs of aflatoxicosis in suckling piglets, swine, dairy and beef cattle, chicken, and turkey poults include decreased feed efficiency, decreased rate of weight gain, nephritis, toxic hepatitis, and systemic hemorrhage [1,6,7]. However, the association in human populations is much more difficult to elucidate, as multiple other risk factors for growth faltering are frequently co-occurring in populations chronically exposed to aflatoxin. Work conducted in Benin and Togo showed that children who were classified as being stunted or underweight had 30–40% higher mean aflatoxin biomarker concentrations [8], and there was a negative correlation (p< 0.0001) between aflatoxin exposure and height increase [9]. In addition, cessation of exclusive breastfeeding and introduction of solid foods have been associated with an increase in aflatoxin exposure [3]. Hence, introduction of solid weaning foods increases child exposure to aflatoxin, during a pivotal time in their development.

Childhood stunting continues to be endemic in particular areas of the world, even following significant feeding and nutrition intervention programs in those areas most affected. A review of nutritional interventions on child growth indicated an increase of 0.7 in height-for-age z-scores (HAZ) to be the highest growth improvement afforded from feeding and nutritional programs [10]. This increase accounts for only one-third of the average growth deficit in children in Asia and Africa [10]. Therefore, other risk factors for growth faltering, such as mycotoxin exposure, are being evaluated, to determine their relative importance in stunting and to design appropriate interventions to improve child growth outcomes.

The Etiology, Risk Factors and Interaction of Enteric Infections and Malnutrition and the Consequences for Child Health and Development (MAL-ED) study was designed to provide epidemiological data on risk and protective factors for child growth and development from eight distinct sites around the world [11]. The MAL-ED researchers followed children from birth to 24 or 36 months of age, collecting data on family socioeconomic status (SES), mother’s education, anthropometrics of children, diarrheal incidence and enteric pathogens, vaccination efficiency, breastfeeding, dietary intakes and micronutrient status. The work described in this manuscript was designed as a supplement to the ongoing MAL-ED study. We hypothesized that aflatoxin exposure could be an important risk factor in deficits in child z-scores and growth trajectories. The World Health Organization (WHO) defines a z-score that is below 2-standard deviations from the mean as wasted (weight for height), stunted (height for age), or underweight (weight for age); or 3 or more standard deviations below the mean as ‘severely’ wasted, stunted, or underweight [12]. For our analysis, the MAL-ED children’s cohort in Bhaktapur, Nepal, was utilized to assess aflatoxin exposure in relation to growth outcomes (z-scores and growth trajectories) and other risk factors for growth impairment.

Nepal has historically had a high stunting prevalence: 37.4% for children under 5-years was reported by the Nepal Central Bureau of Statistics [13]; 42% was reported in Bhaktapur, Nepal, specifically [14]. Ghosh et al. [15] found a stunting and underweight prevalence of about 46 and 52% in children from 6-10-years old. Contamination of cereal grains with aflatoxins has been reported in different locales in the country [16,17]. Preliminary work following pregnant mothers in Nepal showed chronic exposure to aflatoxin during the length of their pregnancy [18]. More data on aflatoxin exposure in Nepal is needed, particularly in children, to estimate the potential role aflatoxin consumption may play in the etiology of growth faltering. In the following cohort study, children were followed from birth to 36 months of age, and assessed for aflatoxin exposure (at three time points) as well as other potential risk factors for growth impairment. The aim of the present work is to assess potential associations between aflatoxin individually and with combined risk factors and growth-related z-scores at different ages.

## Materials and methods

### Study design and participants

A detailed outline of the MAL-ED study design is available from The MAL-ED Network Investigators [11]. Briefly, children were enrolled from the Bhaktapur community within 17 days of birth between May 2010 and February 2012 and followed for the first 24 months of life with a 36-month follow-up visit. The field office for this work was maintained at the Siddhi Memorial Children’s Hospital in Bhaktapur, Nepal. Inclusion criteria for participation included: a mother aged 16 years or older; singleton pregnancy, plan to remain in the study area for at least 6 months following enrollment, and birth weight or enrollment weight of greater than 1500g [11]. Any children with diagnosed congenital disease or severe neonatal disease were excluded. A description of the Nepal study site is contained in Shrestha et al. [19]. Briefly, Bhaktapur is located approximately 15 km southeast of capital city Kathmandu, with over 90% in the sub-tropical climate zone between 1,000–2,000 m in altitude. IRB approval was obtained from the Nepal Health Research Council and Walter Reed Army Institute of Research, USA, and written informed consents were obtained from the parents [11]. In total, plasma samples from 85 children enrolled in the MAL-ED cohort were included in this analysis; plasma samples collected at 15, 24, and 36 months were analyzed for the AFB_1_-lysine (AFB_1_-lys) adduct.

### Aflatoxin plasma biomarker analysis

Aflatoxin exposure in human populations can be determined by collection of blood plasma and analysis for the metabolite AFB_1_-lys. The sensitivity of this biomarker has been documented in the literature [20] and is standard for long-term exposure investigation. AFB_1_-lys concentrations were assessed by isotope dilution mass spectrometry; the full method is described in Groopman et al. [18] and McCoy et al. [21]. Briefly, 200 μl of plasma was mixed with a 10 μl x 0.1 ng AFB_1_-D4-lys/ml internal standard and pronase solution. This solution was incubated for 18 hours at 37°C. Samples were passed through solid-phase extraction columns and resulting eluent injected onto a UPLC with mass spectrometric detection system for analysis. The internal standard parent molecular ion [(M + H)^+^, m/z 461.3] fragmented to yield a daughter ion at m/z 398.2. The AFB_1_-lys molecular ion (m/z 457.2) fragmented to yield a daughter ion at m/z 394.1. Three quality control samples from rats dosed with AFB_1_ were run daily. The limit of detection was 0.4 pg AFB_1_-lys/mg albumin.

### Growth, breastfeeding, micronutrients, and stool measurements

Methods for obtaining growth measures by MAL-ED staff can be found in Richard et al. [22]. Briefly, study staff members were trained to measure anthropometrics of children enrolled in the study on a monthly basis. Several quality control measures were in place at the study site; standardized techniques and instruments were implemented across study sites, and the measurement was repeated on a subset of participants throughout the follow-up for quality assurance purposes. Standard recumbent length measuring boards (Shorrboards, UNICEF), infant scales (seca medical scales), and non-stretch Teflon synthetic tape (seca medical scales) were used to measure length, weight, and head circumference, respectively.

After enrollment, each participant was visited twice per week for data collection on illness, infant feeding practices, and any diarrheal stool samples. Caregivers were questioned about the infant’s breastmilk consumption in the prior 24-hours. Infant feeding practice information collected included consumption of: breast milk, animal milk, formula, water, tea, fruit juice, other liquids, or semi-solids. Patil et al. [23] provides further details on the breastfeeding questionnaires and surveillance. Exclusive breastfeeding was defined as breastfeeding without the introduction of other food or liquids (not even water) since birth, with the exception of delivery systems of vitamins, mineral supplements, or medicine. Predominant breastfeeding was classified as infants receiving (beyond breast milk) plain water or water-based liquids, such as tea or juice. Inclusion of milks, formula, or semi-solid foods was classified as partial breastfeeding [23]. For our analysis, the age at which children were first given semi-solid or solid foods and the age at which weaning was completed were compared with aflatoxin exposure concentrations.

Caulfield et al. [24] provide in depth characterization of the methodology for data collection on infant and child feeding practices. A quantitative 24-hour recall approach was utilized to derive child food and nutrient intake. The 24-hour recalls were conducted each month from 9–36 months. Through combining the individual monthly data over specific time points (9–15, 16–24, and 25–36 months) we were able to calculate more precise intake data for individual nutrients that indicated nutrient intake during our desired time points of interest for aflatoxin (15, 24, and 36 months). In Nepal, infants were most commonly fed small bites of rice dipped in a sauce by hand during weaning. Therefore, field-workers utilized balls of dough to gauge amounts of food delivered by caregivers and detailed recipes were collected for calculation of nutrient intake [24]. The nutrient intake variables used in our current analysis included vitamin A, zinc, and iron. These intake variables were adjusted for total energy intake averaged across the corresponding time points by calculation of the residuals from an ordinary least squares regression analysis. The residual values were utilized in all statistical analysis.

Similarly, number of grain food items consumed per day was assessed through 24-hour food recall. Grain items can include foods containing rice, maize, wheat, millet, etc. These data were averaged by individual participant over 9–15, 16–24, and 25–36 months. The data was normally distributed (goodness of fit test, p<0.2162) and were not transformed or adjusted for statistical analysis.

In addition to consumption calculations, blood samples collected at 15 and 24 months were analysed for haemoglobin, zinc, and vitamin A status. Hemoglobin concentration was measured at the time of blood collection by the HemoCue method [24]. Individual participants were classified dichotomously, as either anemic or not anemic after adjusting for high altitude. An adjusted haemoglobin concentration of <11 gm/dl was classified as anemic. Plasma zinc and plasma retinol concentrations were measured as indicators of zinc and vitamin A status, respectively [24].

Stool sampling and testing methodology can be found in Houpt et al. [25] and Platts-Mills et al. [26]. Biomarkers of gut inflammation and permeability that were collected by the MAL-ED group of investigators included the lactulose-mannitol test, α-1-antitrypsin (ALA), neopterin (NEO), and myeloperoxidase (MPO) [11]. In this work, we assessed the association of aflatoxin with ALA, NEO, and MPO as well as the potential of impaired gut function as a pathway between aflatoxin and impaired growth in the Nepalese population. ALA concentrations were used as an indicator of gut permeability, NEO as an indicator of T-helper 1 immune activation, and MPO as an indicator of neutrophil activity. To test for normality, we evaluated the skewness and kurtosis of each of the gut inflammation variables. Variables with absolute value of skewness greater than 3 or kurtosis greater than 10 were regarded as non-normal [27]^.^ A majority of the gut inflammation and permeability measures were non-normal at the 15, 24, and 36 month time points, and were therefore transformed with natural logarithm prior to statistical analysis.

### Socioeconomic status

Measurement of SES is a theoretical construct that typically attempts to conceptualize an individual, household, or community access to resources. MAL-ED investigators conducted preliminary research to determine those variables and measurements that would be applicable in a multi-country study, and formulated a simple complete index of household SES called the WAMI index [28]. Based on their preliminary study, it was concluded that the WAMI index was a valid index for use across resource poor settings to predict HAZ [28]. The WAMI index includes components of improved access to water and sanitation, wealth measured by a set of eight assets, maternal education, and monthly household income. The approach for combining the components into a complete measure can be found in Paski et al. [28]. These WAMI index scores were the standard SES-based scores used in this analysis.

### Statistical analysis

The AFB_1_-lysine data were not normally distributed, and were log-transformed for statistical analyses. z-scores were calculated by the WHO standards for birthweight, enrollment weight, length-for-age (LAZ), weight-for-age (WAZ), and weight-for-length (WLZ). Height parameters are not normally used for children less than 24 months of age; therefore, we utilize length measurements to have consistency across z-scores over the studied age range (S1 Dataset). Confounding variables such as age, WAMI index, age at introduction of solid foods, age at completion of weaning, mother’s education, and energy adjusted iron, zinc, and vitamin A consumed were investigated for relationships to anthropometric z-scores and AFB_1_-lys. Variables that could potentially lie within the causal pathway of aflatoxin-associated to growth faltering, including gut function variables (MPO, NEO, and ALA), zinc concentration, retinol concentration, and anemia, were considered during pathway analysis. Of all potential confounders, age and energy adjusted iron consumption were significantly associated with LAZ. Energy adjusted zinc consumption was significantly associated with LAZ, WAZ, and WLZ. Multivariate relationships between AFB_1_-lys and z-scores were analyzed by a random effects model for repeated measures with the observed covariates of energy adjusted iron, zinc consumption, and age.

The growth trajectory, assuming linear segments, of the children were also calculated for LAZ and compared with the AFB_1_-lys concentration found at the older age of the difference between LAZ values (e.g. the AFB_1_-lys value used for the trajectory between 15 and 24 months was the 24-month value).

In addition to utilizing the z-score data as continuous, the data were also categorized into one of four ranges to define the mean AFB_1_-lys values by severity of wasting, stunting, and underweight. Z-scores were sorted into one of 4 classes: 1) non-negative z-score, 2) z-score between -0.01 and -2.0, 3) z-score between -2.01 and -3.0, and 4) z-score less than -3.0. AFB_1_-lys concentrations and z-score classes were analyzed by a mixed model due to large differences in number of values within each class.

The aflatoxin relationship with average consumption of grain foods was determined through a random effects model for repeated measures by age 15, 24, and 36 months.

## Results

The LAZ, WAZ, and WLZ values were normally distributed however, a random effect assay for repeated measures indicated statistically significant differences between LAZ and WAZ values across age (**Table 1**). Based on the WHO definition of a stunted, wasted, or underweight z-score (-2 or less) the average child in this cohort was not suffering from impaired growth. The highest prevalence of z-scores ≤ -2 occurred for the LAZ (19%), which is significantly lower than the previously reported prevalence in Nepal. The mean LAZ values were -1.18, -1.35, and -1.22 at 15, 24, and 36 month of age; while the WLZ values were -0.46, -0.27, and -0.28, respectively. The mean WAZ values were -0.91, -0.92, and -0.82 at 15, 24, and 36 months of age, respectively. The average birth weight z-score for children in the cohort was -0.75 with a minimum value of -3.09. Less than 10% of the infants utilized in this analysis reported a low birth weight (<2,500g).

**Table 1:** Descriptive statistics for aflatoxin and z-scores across age.

	Age (months)			p-value
	15	24	36	
Aflatoxin*	3.85 (15.75)	3.05 (4.71)	4.06 (22.72)	0.251
N	77	85	85	
LAZ‡	-1.18 (-1.39, -0.96)	-1.35 (-1.54, -1.16)	-1.22 (-1.42, -1.02)	<0.0001
WAZ‡	-0.91 (-1.13, -0.69)	-0.92 (-1.09, -0.75)	-0.82 (-1.01, -0.63)	0.0001
WLZ‡	-0.46 (-0.69, -0.23)	-0.27 (-0.44, -0.10)	-0.28 (-0.46, -0.10)	0.052

P-values represent differences across age for each variable.

* Geometric mean (SD)

‡Mean (95% CI)

**Fig 1:**
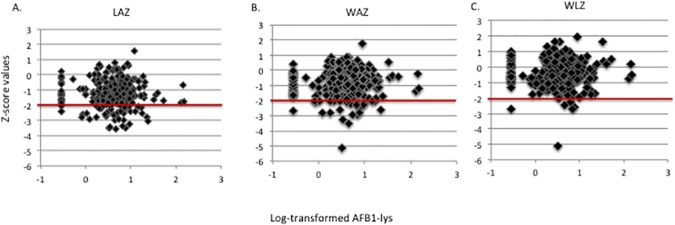
Serum Aflatoxin association with z-scores: Serum AFB_1_-lys level vs. A) LAZ, p = 0.9822; B) WAZ, p = 0.7024; and C) WLZ, p = 0.693. p-values represent those calculated for simple linear regressions. * Clustered points at AFB_1_-lys level of -0.4 represents subjects with AFB_1_-lys levels below the limit of detection. The red line delineates the mark of a z-score ≤ -2, the WHO cutoff for stunting, wasting, or underweight designation.

Neither z-score values (LAZ, WAZ, and WLZ) nor AFB_1_-lys values showed any significant associations among potential confounding variables such as WAMI index, weaning age, mother’s education level, vitamin A plasma concentrations, and consumption of grains. However, as previously discussed, they were associated with age and energy-adjusted iron and zinc consumption. These confounders were not associated with AFB_1_-lys concentration. Regression analysis, including these confounders, of AFB_1_-lys association with z-scores did not reveal any associations. **Fig 1A–1C** shows the linear regression plots of each z-score in relation to the respective AFB_1_-lys value. All of the figures demonstrate a lack of association with the growth parameters and aflatoxin exposure. There were no associations found between AFB_1_-lys and growth trajectory based on the difference in LAZ values across age for 15–24 months, 24–36 months, and 15–36 months.

Weaning status of children was also not associated with AFB_1_-lys concentration. Age of first solid food and completion of weaning were all taken into consideration during the study. The median age of introduction of solid foods was 88 days, and median age at completion of weaning was 754 days. The earliest cessation of weaning occurred at 566 days, while milk, formula, and/or solid food were given as early as 5 days old. The weaning parameters studied here did not have an effect on growth status or AFB_1_-lys concentration. Average consumption of number of grain foods was calculated for each child at a specific age. Mean consumption of grain foods between 9–15 months was 3.4 items per day; at 16–24 months it was 4.0 items; and at 25–36 months the mean was 5.1 items. However, the consumption of grain items were not significantly associated with AFB_1_-lys concentrations.

The MPO concentrations at 15 months were inversely associated with AFB_1_-lys (p-value 0.012), but the NEO and ALA values were not significantly associated with aflatoxin at this time point. Additionally, all of the gut inflammation and permeability biomarkers were not significantly association with AFB_1_-lys at 24 or 36 months of age.

## Discussion

The prevalence of stunting, underweight, and wasting was low in this study population compared with previously reported rates [13–15]. Potential factors affecting the low rate of growth faltering in our population include the study site location, a semi-urban area with higher average income and lower stunting rate; and the age of the children enrolled. Data from Nepal suggests that stunting increases with age; among children 9–11 months stunting has been reported to be 14%, while children that are 36–47 months old have a rate of 53% [29]. Mycotoxin exposure has been proposed as a potential variable contributing to growth deficits and environmental enteric dysfunction [1,30]. Shirima et al. [31] followed 166 children (recruitment age 6–14 months) for 12 months and observed a negative association between fumonisin exposure and LAZ; however, aflatoxin exposure (AFB_1_-alb; ELISA) was not associated with LAZ. The geometric mean for AFB_1_-alb concentrations for this cohort of children was 4.7, 12.9, and 23.5 pg/mg albumin at recruitment, 6, and 12 months after recruitment, respectively. In contrast, a study conducted in Benin, West Africa showed a significant inverse relationship between AFB_1_-alb concentrations and HAZ values when analyzed by quartiles of HAZ [9]. However, children in the lowest HAZ quartile were exposed to AFB_1_-alb values >101.5 pg/mg albumin. Gong et al. [8] also found a significant association between AFB_1_-alb concentration and stunting and underweight, with stunted and underweight children having 30–40% mean higher aflatoxin concentrations. The geometric mean for those children with HAZ or WAZ-scores ≤ -2 or < -3 ranged between approximately 28 and 50 pg/mg albumin.

It is important to note that in all previous aflatoxin/child growth epidemiology work, a different aflatoxin biomarker was measured: aflatoxin B_1_-albumin (AFB_1_-alb). The two biomarkers are based on different methodologies, which use different concentration calculations based on a non-linear (ELISA) versus a linear regression (MS) standard curve. Comparisons between the two methodologies indicate that the AFB_1_-lys marker is approximately 2.6 times more specific [20, 21]. Scholl et al. [20] provided the estimate of 2.6 larger values given by the ELISA method vs. the MS method based on the average difference of paired samples across an AFB_1_-lys concentration range of approximately the limit of detection (0.40 pg/mg albumin) and 23 pg/mg albumin. Although the consistency of this difference between the two methods, particularly at levels exceeding this range is uncertain, the use of the 2.6 factor is currently the best estimate to compare between studies. References between this study and the previous work conducted in Africa should take this factor into consideration when comparing measures of central tendency. The geometric mean value of our population multiplied by 2.6 equals 9.36 pg/mg albumin and the lowest quartile is <2.09 pg/mg albumin and the highest quartile is equal to >19.98 pg/mg albumin. Additionally, the upper quartile mean, multiplied by the 2.6 factor was 52.44 pg/mg albumin.

While the aflatoxin concentrations observed in our Nepalese children’s cohort are within the range observed in the studies from African cohorts, they most closely mimic those from the Tanzania cohort [31, 32] and the Gambian infant trial [33], which did not show a significant correlation with AFB_1_-alb in infants and LAZ. Therefore, it is probable that the exposure in Bhaktapur, Nepal is not high enough, particularly in the upper quartile, to negatively affect growth in children. Additionally, growth effects from chronic aflatoxin exposure, at these low concentrations, may take longer to manifest. Many of these studies accounted for some possible confounding variables such as; gender, age, breastfeeding, village, mother’s education level, SES, and agro-ecological zone, serum retinol, and zinc. In this study we have also included gut inflammation and permeability biomarkers, as well as anemia. There was no correlation with the gut inflammation and permeability biomarkers except for a positive association with MPO and AFB_1_-lys at only one time point (15 months). These results would indicate that aflatoxin did not have more than a minimal effect on gut inflammation in this population.

Aflatoxin exposure in African populations has also typically been associated with weaning status, coinciding with increased consumption of maize- and peanut-based weaning foods [3,34]. In this cohort, there was no association between aflatoxin exposure and weaning status, age, or consumption of grain-based foods. Previous work from the MAL-ED network of investigators has indicated an overall trend of early transition away from exclusive breastfeeding in the first month of life [23]. The mothers in this analysis had one of the highest reported incidences of feeding formula to their infants (15%) and the highest reported consumption of solids/semi-solids by children in the first month of life, out of all eight MAL-ED study sites [23]. Typical weaning foods in the Nepal site included rice prepared and dipped in sauces that differed in composition, depending on ingredient availability [24]. This early introduction of solid foods, that would be the primary source of aflatoxin exposure, could have hindered our ability to detect a shift in exposure due to weaning. The earliest measure of aflatoxin exposure for this study was obtained at 15 months, making it highly probable that by 15 months, the children in the cohort were already at a steady state of low aflatoxin exposure. Additionally, the large intake of rice as the primary grain, which is not a favorable substrate for aflatoxin contamination, would indicate the reason for a lack of association with aflatoxin biomarker level and grain intake variables.

There is evidence in cell and animal models that aflatoxin exposure may compromise gastrointestinal integrity, induce inflammation and permeability [30], and diminish nutrient absorption [35, 36]. The associations of three markers of gut inflammation and permeability–MPO, NEO, and ALA—with aflatoxin exposure were assessed, but no correlations were found in our study other than for an inverse association with MPO and AFB_1_-lys at the 15-month time point.

This study has several limitations. First, some participants did not have a plasma sample at the 15-month time point; hence, we could not determine aflatoxin exposure for those individuals at 15- months. Second, the sample size in this study (85) is smaller than other studies that have been discussed previously and have shown positive associations with stunting. The available number of samples for the analysis of aflatoxin exposure limits the statistical power of the results observed here. Third, the weaning information from Patil et al. [23] indicates that to comprehensively elucidate the effect of weaning on aflatoxin exposure would require that we had aflatoxin biomarker data from the first 3 months of life. General low levels of aflatoxin exposure and low incidence of stunting, wasting, or underweight z-score values among the population tested, compared with those from Benin and Togo, were contributors to the difficulty in finding associations between these variables. Aflatoxin exposure during pregnancy in Nepalese women has been observed [18] and it is well known that aflatoxin can cross the placenta and has been found in cord blood [37]. *In utero* exposures, determined from maternal blood AFB_1_-lys biomarkers, were strongly associated with both reduced weight and length gain in Gambian infants from birth to 52 weeks of age, however child biomarkers were not [33]. The Turner et al. study concluded that a reduction in maternal AFB_1_-alb from 110 pg/mg albumin to 10 pg/mg albumin could lead to a 0.8 kg and 2 cm increase in weight and length, respectively, in the first year of life. Maternal exposure in Nepalese women had a geometric mean 1.5 times lower [18] than the geometric mean observed in pregnant Gambian women [33]. Therefore, future work should be conducted on the consequences of *in utero* aflatoxin exposure on postnatal growth of children.

There are currently 8–10 million deaths of children under age 5 around the world annually, with more than 90% occurring in developing countries [38]. Black et al. [39] estimates that maternal and child undernutrition is the underlying cause of 3.5 million of those deaths. Childhood stunting is associated with increased risk of child morbidity and mortality [40] and impaired cognitive development [41, 42]. The historical high incidence of growth faltering in Nepal and evidence for aflatoxin exposure made this site an ideal location for analysis of potential associations with aflatoxin exposure and growth outcomes. Additionally, all previous associations of aflatoxin exposure and underweight, stunting, and/or wasting incidence have been conducted in populations of sub-Saharan Africa. Here, we analyzed these potential associations in a geographically and ethnically different cohort. The objective of the current work was to examine any potential role of aflatoxin exposure in the network of variables contributing to growth impairment in populations of developing countries. The results from this work, and previous epidemiology studies conducted in Africa, indicate that the effect of aflatoxin on growth outcomes could involve a threshold of effect. Future aflatoxin-related research that includes child stunting as the health endpoint of interest should take into account multiple confounders of effect, and would ideally be conducted across multiple ethnically and culturally diverse populations to determine a threshold value.

## Supporting information

S1 Dataset Nepal child dataset of aflatoxin and z-scores. (XLSX)
